# Uncovering the Pharmacological Mechanism of 2-Dodecyl-6-Methoxycyclohexa-2,5 -Diene-1,4-Dione Against Lung Cancer Based on Network Pharmacology and Experimental Evaluation

**DOI:** 10.3389/fphar.2021.617555

**Published:** 2021-02-02

**Authors:** Lihui Wang, Xin Yang, Qiong Song, Jiejun Fu, Wenchu Wang, Kechen Du, Shuai Chen, Jinjin Cao, Renbin Huang, Chunlin Zou

**Affiliations:** ^1^ Key Laboratory of Longevity and Aging-related Diseases of Chinese Ministry of Education, Center for Translational Medicine and School of Preclinical Medicine, Guangxi Medical University, Nanning, China; ^2^ Department of Pharmacology, Guangxi Medical University, Nanning, China

**Keywords:** DMDD, lung cancer, network pharmacology, cell cycle arrest, TCGA

## Abstract

**Background:** 2-Dodecyl-6-Methoxycyclohexa-2, 5-Diene-1,4-Dione (DMDD) was purified from the roots of *Averrhoa carambola L.* Previous research demonstrated that DMDD is a small molecular compound with significant therapeutic potential for tumors. However, the potential targets and pharmacological mechanism of DMDD to treat lung cancer has not been reported.

**Methods:** We employed network pharmacology and experimental evaluation to reveal the pharmacological mechanism of DMDD against lung cancer. Potential therapeutic targets of DMDD were screened by PharmMapper. Differentially expressed genes (DEGs) in The Cancer Genome Atlas (TCGA) lung cancer data sets were extracted and analyzed by GEPIA2. The mechanism of DMDD against lung cancer was determined by PPI, gene ontology (GO) and KEGG pathway enrichment analysis. Survival analysis and molecular docking were employed to obtain the key targets of DMDD. Human lung cancer cell lines H1975 and PC9 were used to detect effects of DMDD treatment *in vitro.* The expression of key targets after DMDD treated was validated by Western Blot.

**Results:** A total of 60 Homo sapiens potential therapeutic targets of DMDD and 3,545 DEGs in TCGA lung cancer datasets were identified. Gene ontology and pathway analysis revealed characteristic of the potential targets of DMDD and DEGs in lung cancer respectively. Cell cycle and pathways in cancer were overlapping with DMDD potential targets and lung cancer DEGs. Eight overlapping genes were found between DMDD potential therapeutic targets and lung cancer related DEGs. Survival analysis showed that high expression of DMDD potential targets *CCNE1* and *E2F1* was significantly related to poor patient survival in lung cancer. Molecular docking found that DMDD exhibited significant binding affinities within the active site of CCNE1 and E2F1. Further tests showed that DMDD inhibited the proliferation, migration and clone formation in lung cancer cell lines (H1975 and PC9) in a dose and time dependent manner. Mechanistically, DMDD treatment decreased the expression of CDK2, CCNE1, E2F1 proteins and induced cell cycle arrest at the G1/S phase in H1975 and PC9 cells.

**Conclusion:** These results delineated that DMDD holds therapeutic potential that blocks tumorigenesis by cell cycle regulation in lung cancer, and may provide potential therapies for lung cancer.

## Introduction

Lung cancer is the most frequently diagnosed and leading cause of cancer-related deaths globally (Siegel et al., 2020), with the most common subtypes being adenocarcinomas (AD) and squamous cell carcinomas (SCC) (Youlden et al., 2008). Since there were no obvious clinical symptoms or only mild symptoms in the early stage of lung cancer, nearly 60–80% lung cancer patients were in the advanced stage when the diagnosis was confirmed and missed the best time for surgery (Motoi et al., 2008; Scagliotti et al., 2008; Kowalczyk and Jassem, 2020). In order to improve the patient's life quality and prolong their survival time, chemotherapy has become one of the main treatments for advanced lung cancer besides surgical treatments. At present, the first-line chemotherapy for lung carcinoma treatment is platinum-based drugs combined with third-generation chemotherapy drugs (Scagliotti et al., 2008; Li et al., 2020; Yao et al., 2020). Because of serious side effect and multi-drug resistance, many patients with lung cancer are forced to stop drug treatment. There is an urgent need for new, effective, safe and low toxic antitumor drugs for lung cancer.

Searching for the compounds with high efficiency and low toxicity from natural products is one of the main research strategies for antitumor drugs (Tariq et al., 2017). Around 49% small molecules for cancer chemotherapy are natural products (Newman and Cragg, 2016) (Alves-Silva et al., 2017; Seca and Pinto, 2018). The anticancer effects of natural products and their secondary metabolites are widely used in the treatment of tumors, including paclitaxel, vincristine, etoposide and camptothecin (Ouyang et al., 2014). 2-dodecyl-6-methoxycyclohexa-2, 5-diene-1,4-dione (DMDD), an active quinonoids from the roots of *Averrhoa carambola L.*, exhibits various biological effects, including antidiabetic (Kintoko et al., 2018), antidiabetic nephropathy (Lu et al., 2019) and anti-breast cancer (Gao et al., 2015; Chen et al., 2017; Zhou et al., 2020) activities. The various biological activities and low toxicity of DMDD make it a promising candidate for clinical development. Recent studies have found that the cytotoxic effects of DMDD against human breast, lung and bone cancer cells *in vitro* (Gao et al., 2015). However, the potential targets and pharmacological mechanism underlying DMDD regulated lung cancer tumorigenesis still remain unrevealed.

In recent years, network pharmacology and bioinformatics analysis has provided new ideas and new solutions for studying the mechanism of natural products (Kibble et al., 2015; Poornima et al., 2016; Liu et al., 2019). In order to uncover the comprehensive mechanisms of DMDD, we adopted a systematic analysis based on network pharmacology to screen out the potential targets and functional characteristics of DMDD for lung cancer treatment. At the same time, we screened out potential targets of DMDD differentially expressed and associated with poor patient outcomes in lung cancer. These results were further verified by *in vitro* experiments. We have identified that E2F1 and CCNE1 which are DMDD potential targets, were upregulated in lung cancer. Clinical studies have shown that high expression of *E2F1* and *CCNE1* are associated with poor prognosis in lung cancer patients. *In vitro* experiments showed that DMDD is an effective inhibitor of E2F1 and CCNE1 and leading to the suppression of proliferation, migration and clone formation in lung cancer cells. Mechanistically, DMDD treatment decreased the expression of CDK2, CCNE1, E2F1 proteins and up-regulated the p21 proteins, as well as induced cell cycle arrest at the G1/S phase in H1975 and PC9 cells. This study explored the targets and key signaling pathway of DMDD for lung cancer, providing potential strategies for the treatment of lung cancer.

## Materials and Methods

### DMDD Targets Prediction

To identify the potential targets of DMDD, the drug target predictions database PharmMapper (http://lilab-ecust.cn/pharmmapper/) was carried out. The PharmMapper database (Liu et al., 2010; Wang et al., 2016; Wang et al., 2017) was designed to identify potential targets for small molecules using pharmacophore mapping approach. DMDD MOL2 format file was uploaded into the web-server and the DMDD potential targets were obtained and sorted by normalized fit score value. Only the Homo sapiens protein targets were selected.

### DEGs Analysis

GEPIA2 (Gene Expression Profiling Interactive Analysis 2, http://gepia2.cancer-pku.cn/#index) is a web-based tool to deliver fast and customizable functionalities based on TCGA datasets. 1,078 non small cell lung cancer (NSCLC) samples form TCGA datasets were selected, including 483 lung adenocarcinoma (LUAD) and 59 corresponding normal, 486 lung squamous cell carcinoma (LUSC) and 50 corresponding normal samples. Differentially expressed genes (DEGs) between the NSCLC and normal samples were analyzed using GEPIA2 by LIMMA methods. Using |log2 fold change| ≥ 1 and Q-value < 0.01 as cutoff conditions.

### PPI Network Construction and Functional Enrichment Analysis

To explore the interaction between DMDD targets or DEGs in lung cancer, the DMDD targets or DEGs were analyzed using STRING database (https://string-db.org/) to generate PPI network. PPI pairs with a combined score >0.4 were extracted. The PPI network was visualized using Cytoscape 3.7.2 software (Shannon et al., 2003) and the most important module was performed using the MCODE plug-in in Cytoscape software. In addition, Metascape web-based tool (https://metascape.org/gp/index.html) (Zhou et al., 2019) was used for functional enrichment analysis (GO analysis and KEGG pathway analysis) of these genes.

### Kaplan–Meier Test

Survival information of the DEGs in lung cancer patients were obtained from the Kaplan-Meier Plotter (http://kmplot.com/analysis/), which was established based on the microarray and RNA-seq datasets for several cancer types including lung cancer (Gyorffy et al., 2013). The information of 1,715 lung cancer patients were collected from the Gene Expression Omnibus (GEO, http://www.ncbi.nlm.nih.gov/geo/) (GSE4573, GSE14814, GSE8894, GSE19188, GSE3141, GSE31210, GSE29013 and GSE37745), the Cancer Biomedical Informatics Grid (caBIG, http://cabig.cancer.gov/) caArray and The Cancer Genome Atlas (TCGA, http://cancergenome.nih.gov) NSCLC cohort. Among the 1,715 NSCLC samples, overall survival (OS) data were available for 1,577 samples. Kaplan–Meier analysis was used to plot the OS survival curves. Survival differences were assessed by the log-rank test using the median of DEGs as cutoff value. The hazard ratio (HR) with 95% confidence intervals and log-rank *p*-values were calculated.

### Molecular Docking

The 3D formats of DMDD was obtained from ZINC15 drug database (http://zinc15.docking.org/) (Sterling and Irwin, 2015). The X-ray crystal structure of DEGs were obtained from the RCSB PDB protein databank (http://www.rcsb.org/). The water molecules and ligands molecules were removed from the crystal structures by Discovery Studio 4.5 Client before the docking simulation began. Docking simulations of DMDD and DEGs were carried out by PyRx software. Interaction energies were calculated for predicting docking positions and selecting the binding pose that had the lowest binding energy (kcal mol^−1^). Results was visualized and analyzed using Discovery Studio 4.5 Client.

### Plant Material and Isolation of DMDD

2-Dodecyl-6-Methoxycyclohexa-2, 5-Diene-1,4-Dione (DMDD) was purified from the roots of *Averrhoa carambola L.* as described in our previous study (Wen et al., 2012). The plant material *Averrhoa carambola L.* was collected from Lingshan, Guangxi, China and was identified by Prof Maoxiang Lai (Traditional Chinese Medicine Research Institute of Guangxi). A 20 mM stock solution of DMDD was prepared using dimethyl sulfoxide (DMSO).

### Cell Culture

Human lung cancer cell lines H1975 (ATCC, CRL-5908) were obtained from American Type Culture Collection and maintained using standard media and conditions. PC9 was kindly provided by Dr. Guoan Chen (School of Medicine, Southern University of Science and Technology). H1975 and PC9 were maintained in RPMI 1640 supplemented with 10% FBS and 1% penicillin/streptomycin at 37°C in a 5% CO2 cell culture incubator. Mycoplasma contamination was excluded in these cell lines.

### Cell Viability Assay

Cell viability was detected by CellTiter 96^®^ AQueous One Solution Cell Proliferation Assay (Promega) according to manufacturer's instructions. H1975 and PC9 cells (4 × 10^3^ cells/well) were treated with DMDD at different time and different concentration, and then incubated with CellTiter 96^®^ AQueous One Solution reagent at 37°C in a humidified with 5% CO_2_ for 1h. The absorbance at 490 nm was recorded using an ELISA plate reader. The cell viability rates were calculated by normalizing to the control group.

### Clone Formation Assay

Human lung cancer cell lines H1975 and PC9 (100 cells/well) were seeded into 12-well plates and cultured overnight. Cells were then treated with DMDD at different concentration (0, 10, 20, 40 µM). After 2 weeks, cells were fixed with 4% paraformaldehyde and stained with 0.1% crystal violet. The number of clones was counted in each well.

### Wound Healing Assay

H1975 and PC9 (4.5 × 10^5^ cells/well) were seeded into 12-well plates and cultured overnight. Cells were grown to confluency in a monolayer and treated with DMDD at different concentration (0, 10, 20, 40 µM). A scratch was made with a pipette tip to create an incision-like gap. The scratch area was photographed immediately after wounding and at 24 h time points thereafter, and cell migration was quantified and expressed as average percentage of closure of the scratch area.

### Western Blot

For Western blot analysis, cell lysates were boiled in sample buffer at 95°C for 5 min. The samples were separated by polyacrylamide gel electrophoresis at 90 V for 2 h and transferred onto polyvinylidene difluoride membranes. After blocking for 1 h with 5% non-fat milk, the membranes were incubated with primary antibodies against human CCNE1 (1:1,000 dilution), E2F1 (1:1,000 dilution), CDK2 (1:1,000 dilution), p21 (1:1,000 dilution), GAPDH (1:1,000 dilution) at 4°C overnight. After incubation with secondary antibody (1:2000 dilution) for 1 h at room temperature, the membranes were developed using enhanced chemiluminescence (ECL), and the signals were detected by MiniChemi 610 Plus (SageCreation Science Co.).

### Flow Cytometry Analysis

Forty Eight hours after treatment with DMDD at various concentration (0, 5, 20 µM), H1975 and PC9 cells were harvested and fixed with 70% ice-cold ethanol for 2 h at 4°C, washed with PBS, re-suspended in 1 ml of propidium iodide (PI) staining solution (40 μg/ml RNaseA and 10 μg/ml PI in PBS), and then incubated in the dark at room temperature for 30 min. Samples were transfered to the flow cytometer and used to measure the cell cycle by FACScan Flow Cytometry (BD FACSCalibur).

### Statistical Analysis

Data were analyzed using GraphPad Prism 5 and SPSS 19.0 software. To identify gene expression patterns, an unsupervised hierarchical cluster analysis using un-centered average linkage was performed using Cluster v3.0 after median—centering genes and arrays. Heat maps were visualized using Tree View software. The other data such as proliferation were evaluated by unpaired Student's t-test. A two-tailed *p* value <0.05 was considered significant. To determine potential underlying biological processes associated with DMDD potential targets or DEGs in lung cancer, Gene Ontology enrichment analysis was performed using Metascape web-based tool (https://metascape.org/gp/index.html).

## Results

### Identification and Functional Analysis of DMDD Potential Target Proteins

**FIGURE 1:**
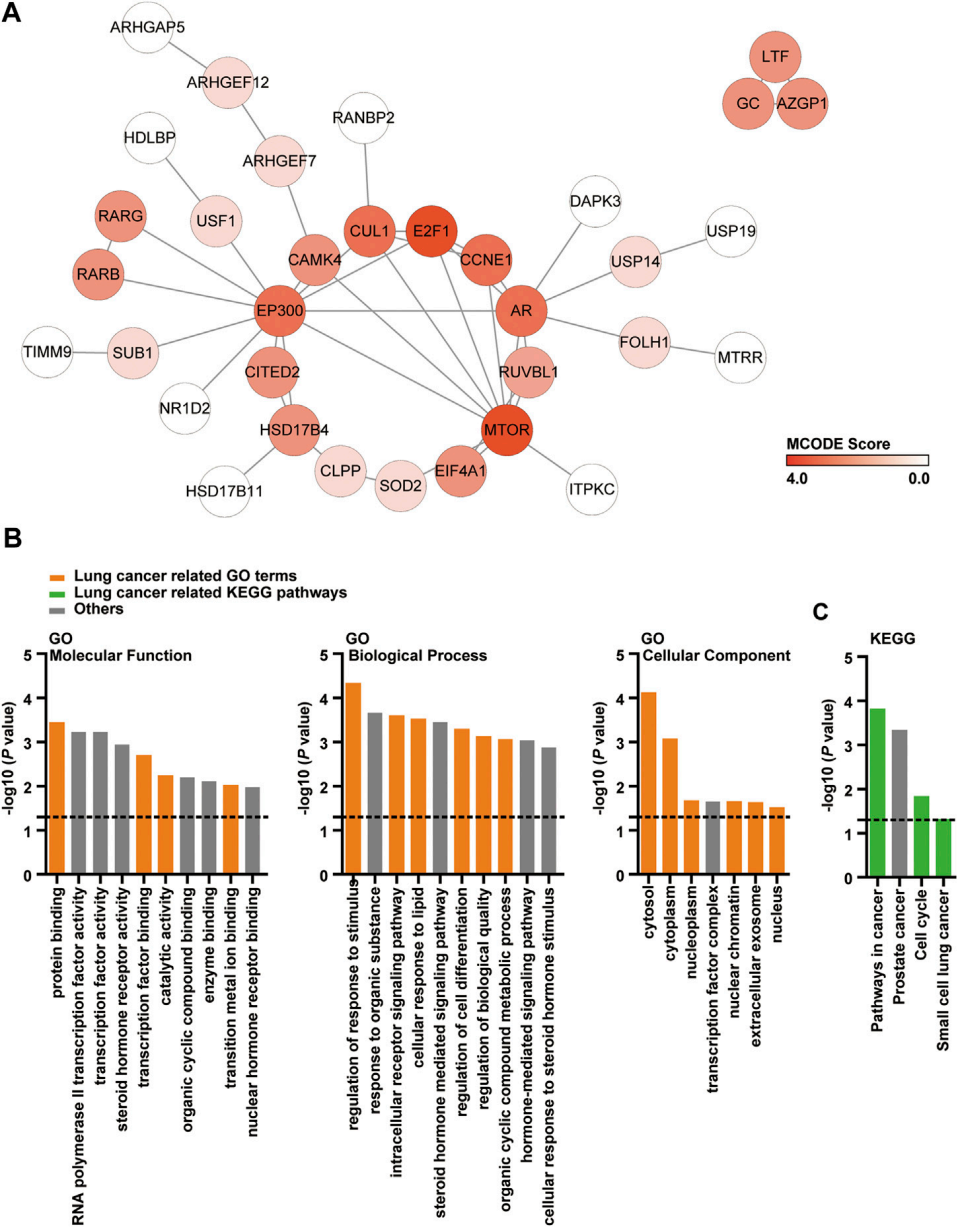
PPI, GO and KEGG analysis of DMDD potential target proteins. **(A)** PPI network of 60 DMDD potential targets which consists of 59 nodes and 47 edges. The node color changes from light to dark red in according to the MCODE score. **(B, C)** Cellular component, molecular function, biological process, and KEGG analysis of 60 DMDD potential target proteins.

We predicted the DMDD potential target proteins and found 60 candidates (Supplementary Table S1). PPI networks of these 60 potential targets was subsequently analyzed. The PPI network contained 59 nodes and 47 edges which indicated that there was a wide range of interactions among these proteins (Figure 1A). MCODE algorithm results showed that MTOR (MCODE Score = 3.47), E2F1 (MCODE Score = 3.47), AR (MCODE Score = 2.7), CCNE1 (MCODE Score = 2.7), CUL1 (MCODE Score = 2.7) and EP300 (MCODE Score = 2.7) were densely connected (Figure 1A). GO enrichment analysis and KEGG pathway analysis were applied to 60 DMDD potential target proteins to further investigate their functions. These proteins were found to be highly involved in specific cellular processes such as protein binding (*p* = 0.00037), regulation of response to stimulus (*p* = 0.000047) (Figure 1B). Pathway enrichment showed that various pathways especially pathways in cancer (*p* = 0.00016), cell cycle (*p* = 0.015) and small cell lung cancer (*p* = 0.049) were involved in DMDD potential target proteins (Figure 1C). These data suggested that cancer related genes and pathways were abundant in DMDD potential targets.

### Identification of Differentially Expressed Genes in Lung Cancer

**FIGURE 2:**
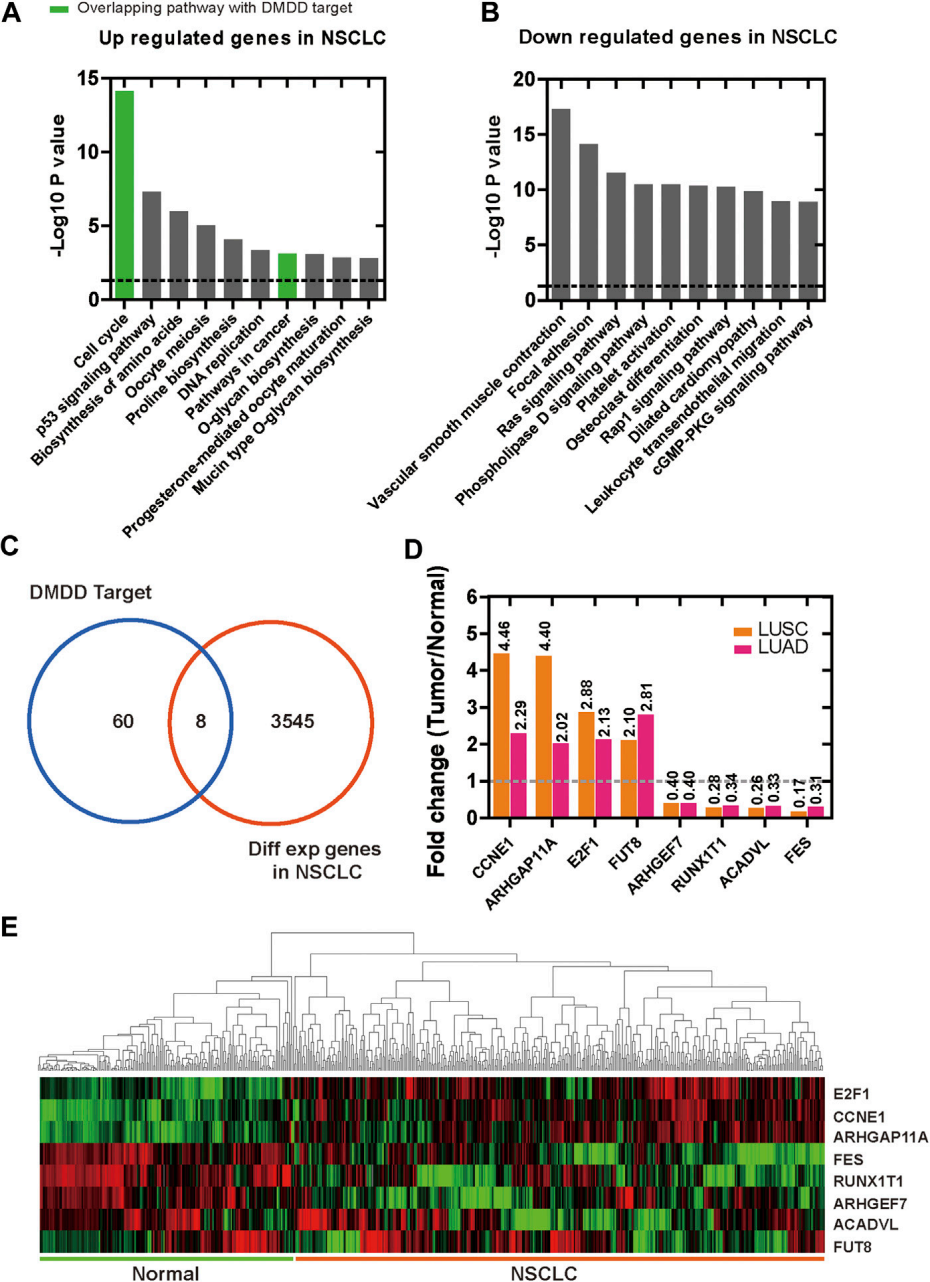
Differentially expressed genes in lung cancer and normal lung tissues. **(A)** Enrichment of KEGG pathways for up regulated genes in lung cancer. **(B)** Enrichment of KEGG pathways for down regulated genes in lung cancer. **(C)** 8 overlapping genes were found between 60 potential therapeutic target proteins of DMDD and lung cancer related DEGs. **(D, E)** *FUT8*, *CCNE1*, *E2F1*, *ARHGAP11A* were upregulated and *ARHGEF7*, *RUNX1T1*, *ACADVL*, *FES* were down regulated in lung cancer tissues compared with normal lung.

We analyzed the DEGs in NSCLC dataset of TCGA and resolved 3,545 candidates, among which 787 were up-regulated and 2,758 were down-regulated in tumor compared with normal tissues (Supplementary Table S2). To further analyses upregulated and downregulated DEGs in lung cancer, we performed KEGG pathway enrichment analysis by using Metascape respectively and use *p* < 0.05 as the cut-off criterion. For upregulated DEGs, the top 3 KEGG pathway were cell cycle, p53 signaling pathway and biosynthesis of amino acids (Figure 2A). Among them, cell cycle and pathways in cancer overlapped with DMDD potential targets KEGG pathway (Figure 2A). For downregulated DEGs, the top 3 relevant KEGG pathway were vascular smooth muscle contraction, focal adhesion and Ras signaling pathway (Figure 2B). Eight overlapping genes were found between potential therapeutic targets of DMDD and lung cancer related DEGs (Figure 2C). The up regulated genes were *FUT8* (FC = 2.10 in LUSC, FC = 2.81 in LUAD, *q* < 0.01)*, CCNE1* (FC = 4.46 in LUSC, FC = 2.29 in LUAD, *q* < 0.01)*, E2F1* (FC = 2.88 in LUSC, FC = 2.13 in LUAD, *q* < 0.01)*, ARHGAP11A* (FC = 4.40 in LUSC, FC = 2.02 in LUAD, *q* < 0.01), and the down regulated genes were *ARHGEF7* (FC = 0.40 in LUSC, FC = 0.40 in LUAD, *q* < 0.01)*, RUNX1T1* (FC = 0.28 in LUSC, FC = 0.34 in LUAD, *q* < 0.01)*, ACADVL* (FC = 0.26 in LUSC, FC = 0.33 in LUAD, *q* < 0.01)*, FES* (FC = 0.17 in LUSC, FC = 0.31 in LUAD, *q* < 0.01) (Figures 2D,E).

### Survival Analysis

**FIGURE 3:**
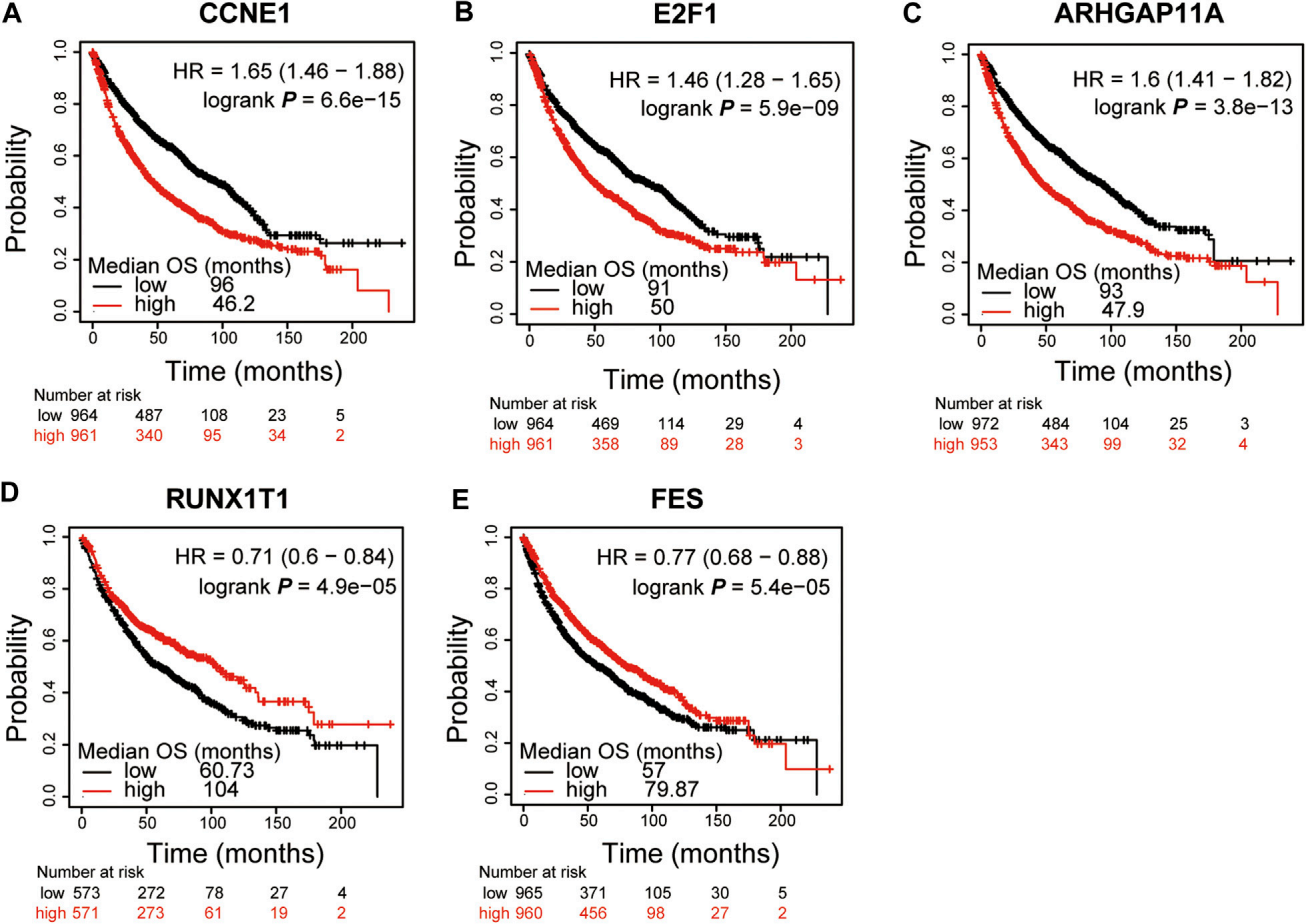
*CCNE1, E2F1, ARHGAP11A, RUNX1T1* and *FES* were significantly associated with patient survival in lung cancer. Kaplan-Meier curves and log-rank test of *CCNE1* **(A)**, *E2F1* **(B)** and *ARHGAP11A* **(C)** in lung cancer data sets indicating higher *CCNE1, E2F1* and *ARHGAP11A* expression were associated to poorer patient survival. Kaplan–Meier curves and log-rank test of *RUNX1T1* **(D)** and *FES* **(E)** in lung cancer data sets indicating lower *RUNX1T1* and *FES* expression were associated to poorer patient survival.

To explore the prognostic value of these 8 DEGs which were also the potential targets of DMDD in lung cancer, the Kaplan Meier plotter online database (Nagy et al., 2018) was used. The results showed that *CCNE1, E2F1, ARHGAP11A, RUNX1T1* and *FES* were significantly associated with patient survival in lung cancer (*n* = 1925). We found that high expression of *CCNE1* (low vs. high: 96.0 vs. 46.2, *p* < 0.01) (Figure 3A), *E2F1* (low vs. high: 91.0 vs. 50.0, *p* < 0.01) (Figure 3B) and *ARHGAP11A* (low vs. high: 93.0 vs. 47.9, *p* < 0.01) (Figure 3C) and low expression of *RUNX1T1* (low vs. high: 60.7 vs. 104.0, *p* < 0.01) (Figure 3D), *FES* (low vs. high: 57.0 vs. 79.9, *p* < 0.01) (Figure 3E) were significantly related to poor patient survival (*p* < 0.01). Then we analyzed the correlation between transcription level of *CCNE1*, *E2F1*, *ARHGAP11A*, *RUNX1T1*, *FES* and tumor stage in TCGA NSCLC cohort by GEPIA 2 online website. The results demonstrated that the expression levels of *CCNE1*, *E2F1*, *ARHGAP11A*, *RUNX1T1* and *FES* displayed significant correlation with the tumor stage in patients with NSCLC (Supplementary Figures S5A–E). These results suggest that *CCNE1, E2F1, ARHGAP11A, RUNX1T1* and *FES* were significantly associated with the tumor stage and can be used as prognostic biomarkers for lung cancer. It's worth noting that *E2F1* and *CCNE1* had high MCODE score in DMDD targets PPI and both of them were demonstrated to be associated with cell cycle and pathways in cancer and small cell lung cancer (Figure 1A).

To explore the transcription levels of *CCNE1, E2F1* in other types of cancer, we profiled the tissue-wise expression of these genes in different cancer types by GEPIA 2 based on TCGA datasets. We found that high expression (log2FC > 1, *q* < 0.01) of *CCNE1* was presented in 18 of 31 cancer types. The top 3 were uterine carcinosarcoma (FC = 45.16, *q* < 0.01), lymphoid neoplasm diffuse large B-cell lymphoma (FC = 43.90, *q* < 0.01) and uterine corpus endometrial carcinoma (FC = 30.08, *q* < 0.01) (Supplementary Figure S1). *E2F1* was significantly high expressed (log2FC > 1, *q* < 0.01) in 22 of 31 cancer types in TCGA data set. Top 3 are lymphoid neoplasm diffuse large B-cell lymphoma (FC = 21.26, *q* < 0.01), liver hepatocellular carcinoma (FC = 19.61, *q* < 0.01), cervical squamous cell carcinoma and endocervical adenocarcinoma (FC = 18.42, *q* < 0.01) (Supplementary Figure S2). Then the correlation analysis of DNA methylation level and transcript level of *CCNE1* and *E2F1* in 370 lung squamous cell carcinoma and 456 lung adenocarcinoma tissues from TCGA cohort was performed to identify the possible mechanism underlying *CCNE1* and *E2F1* DNA methylation in NSCLC by cBioPorta. The results showed that the level of DNA methylation was negatively related to the decreased *CCNE1* and *E2F1* transcription in 370 lung squamous cell carcinoma tissues (*p* < 0.05, Supplementary Figures S3A and S4A) and 456 lung adenocarcinoma tissues (*p* < 0.05, Supplementary Figures S3B and S4B). Indicated that decreased methylation levels of *CCNE1* and *E2F1* might associated with the upregulation of *CCNE1* and *E2F1* in NSCLC tissues.

These results suggest that *CCNE1, E2F1* are involved in the development of various cancers and are closely related to cell cycle and pathways in cancer, etc. Furthermore, decreased methylation levels might associated with the upregulation of *CCNE1* and *E2F1* in NSCLC tissues. Based on the above analysis, we predict that *CCNE1* and *E2F1* holds the potential to be diagnostic/prognostic markers of NSCLC and DMDD might inhibit the development of lung cancer by targeting CCNE1 and E2F1.

### Molecular Docking Verification

**FIGURE 4:**
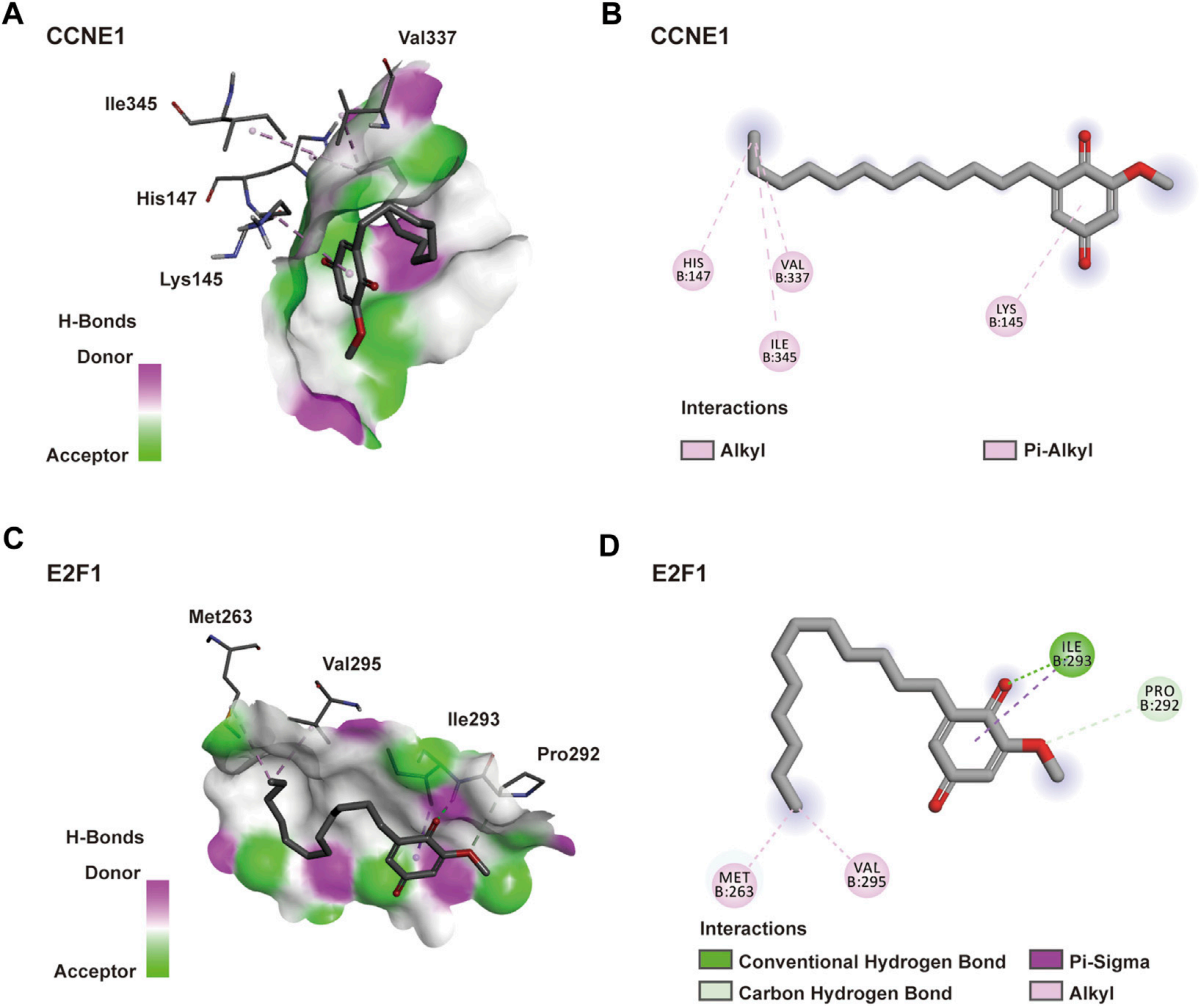
Docking model of DMDD with CCNE1 and E2F1. **(A)** DMDD binding with the pocket of CCNE1 is composed of hydrogen bonds. And the interaction pattern of DMDD with the residues. **(B)** 2D diagram between the CCNE1 and residues. **(C)** DMDD binding with the pocket of E2F1 is composed of hydrogen bonds. And the interaction pattern of DMDD with the residues. **(B)** 2D diagram between the E2F1 and residues.

A molecular docking model was constructed to further explore the mechanism of interaction between DMDD and its potential target proteins. Processing of the DMDD included energy minimized. The refinement of structure of DMDD was used for the dock. PyRx was used for the docking studies. The docked conformation corresponding to the lowest binding energy was selected as the most probable binding conformation. We found that DMDD could enter into the N-terminal cyclin box of CCNE1 (PDB code: 1W98) which leads to pCDK2 kinase activation (Honda et al., 2005), and stayed in the binding pocket surrounded by key residues (His147, Ile345, Val337 and Lys145) (Figure 4A). DMDD could bound with the marked-box domain of E2F1 (PDB code: 2AZE) which leads to Rb and E2F1 binding (Hallstrom and Nevins, 2003), and stayed in the binding pocket surrounded by key residues (Met263, Pro292, Ile293 and Val295) (Figure 4B). Our investigations revealed that DMDD exhibited significant binding affinities within the active site of CCNE1 and E2F1. DMDD may be used as an effective anti-lung cancer drugs by targeting CCNE1 and E2F1.

#### DMDD Inhibits Proliferation, Migration and Colony Formation in Lung Cancer Cells

**FIGURE 5:**
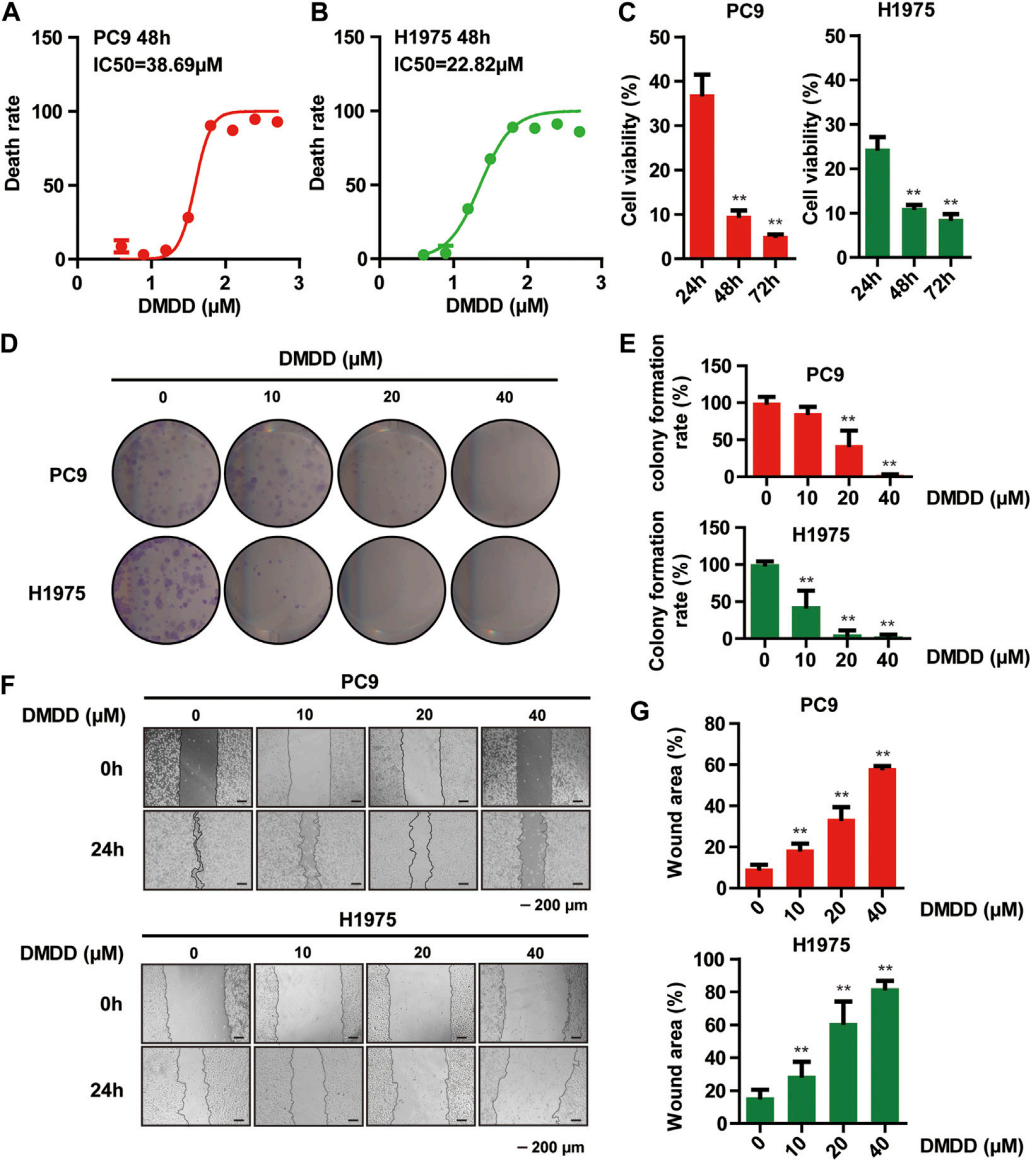
DMDD Inhibits proliferation, migration and colony formation in lung cancer Cells. **(A**, **B)** Dose-dependent curves of DMDD in PC9 and H1975 cell lines. **(C)** Cell proliferation decreased after 60 µM DMDD treatment in PC9 and H1975 cell lines at indicated time. **(D)** Colony formation in PC9 and H1975 cells after treatment with DMDD at 0, 10, 20 and 40 µM. **(E)** Bar chart shows the colony formation rate calculated from Figure 5D. **(F)** Cell migration was determined using a wound-healing assay. Images of the wound areas were shown at 0 and 24 h in PC9 and H1975 cell lines (scale bar 200 μm). **(G)** Bar chart shows the wound area calculated from Figure 5F. Data are shown as mean ± SD. *p < 0.05, **p < 0.01.

To test if DMDD has protective effect against lung cancer, we measured cell proliferation and colony formation followed by DMDD treatment in 2 lung cancer cell lines, PC9 and H1975. Cell proliferation was performed by using MTS assay. The results showed that cell viability of PC9 and H1975 was dramatically decreased by DMDD treatment in a dose and time dependent manner (*p* < 0.05, Figures 5A–C). Similarly, the colony formation was significantly decreased by DMDD treatment in PC9 and H1975 cell lines in a dose dependent manner (*p* < 0.05, Figures 5D,E). Cell migration is an important aspect of cancer progression. Wounding healing assay was performed to investigate the potential effects of DMDD on cell migration. The results showed that cell migration was inhibited by 60% in PC9 and 40% in H1975 after 40 µM of DMDD treatment for 24 h. The inhibitory effect of DMDD on lung cancer cell migration is also dose dependent (*p* < 0.05, Figures 5F,G). These results suggested that DMDD has inhibitory effect on cell proliferation, migration and colony formation in lung cancer cells.

#### DMDD Induces Cell-Cycle Arrest at G1/S via E2F1 and CCNE1 Regulation in Lung Cancer Cells

**FIGURE 6:**
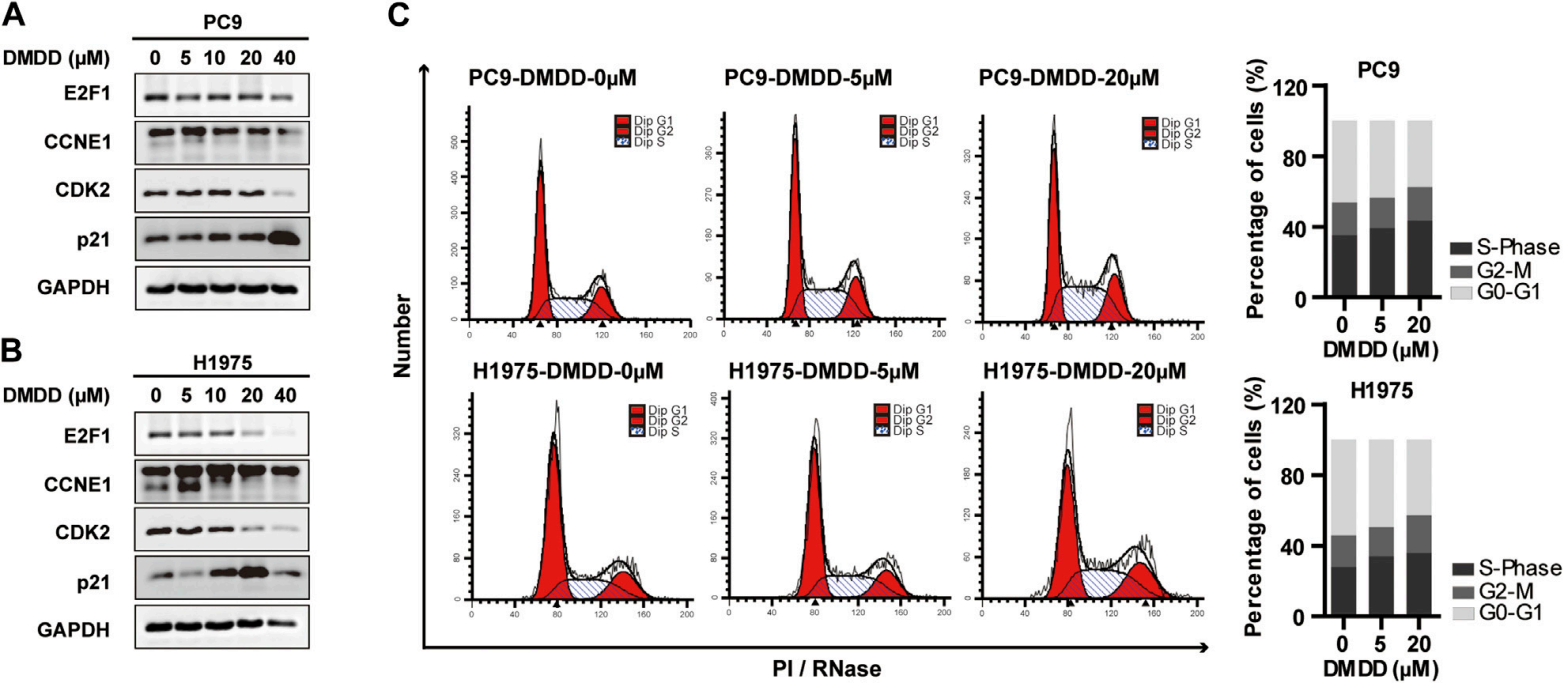
DMDD induced cell cycle arrest at the G1/S phase and potentially through CCNE1 and E2F1 regulation. **(A**, **B)** CCNE1, E2F1 and CDK2 were decreased after DMDD treatment with indicated doses for 48 h measured by Western blot in PC9 and H1975 cells. **(C)** DMDD treatment with indicated doses caused G1/S arrest in both PC9 and H1975 lung cells by flow cytometer.

The above results indicated that DMDD potential targets CCNE1 and E2F1 are closely related to cell cycle. Thus, we performed flow cytometry analysis to investigate whether DMDD affects cell growth through cell cycle regulation. The result showed that 48 h treatment of DMDD induced cell cycle arrest at the G1/S phase (Figure 6C). In order to investigate potential genes regulated by DMDD, we performed Western Blot after 48 h treatment of DMDD in PC9 and H1975 cell lines. Western Blot showed that the expression of CCNE1, E2F1 and CDK2 were significantly downregulated after DMDD treatment while expression of the tumor suppressor gene p21 was significantly increased in H1975 and PC9 cells in a dose dependent manner (Figure 6A,B). Collectively, these results indicate that DMDD induces G1/S cell cycle arrest by regulating its potential targets E2F1 and CCNE1 in lung cancer cancer cells.

## Discussion

For many complex diseases with high incidence in the population, such as diabetes, angiocardiopathy, arthritis and cancer, their pathogenesis are not only attributed to mutation or dysfunction of one single gene, but also caused by dysfunction of whole regulation network (Biankin et al., 2015; Gillet et al., 2009). Previous research for new drugs that target single gene or protein may overlook important therapeutic opportunities (Keith et al., 2005). Drug development for lung cancer face the same challenges. Network pharmacology is one of the strategies for discovering new drugs (Hopkins, 2008). It's a new subject which integrates pharmacology, systems biology, network biology, bioinformatics and other related scientific subjects (Kibble et al., 2015). Network pharmacology tries to reveal the principle of drug action from the point of biological network, and helps to find drug targets and improve drug efficacy (Poornima et al., 2016; Zhang et al., 2019). In this study, the potential therapeutic targets and pathways of DMDD was found by network pharmacology, and the mechanism of DMDD against lung cancer was investigated. As this study has shown, benefits from advances in technology, genomic resources and analytical tools, network pharmacology provides a new idea for understanding the drug’s pharmacological mechanisms in the network perspective.

Over the past decade, natural products have been the mainstay of cancer chemotherapy (Desai et al., 2008; Newman and Cragg, 2016; Alves-Silva et al., 2017). *Averrhoa carambola L.* (Oxalidaceae) root have been commonly used as a traditional Chinese medicine for treating chronic headache, arthralgia and dyspepsia (Committee, 1999). In previous study, an active compound DMDD was separated from the roots of *Averrhoa Carambola L.* (Oxalidaceae) and observed to contain several biological properties (Ouyang et al., 2014). Previous studies have suggested that DMDD shows significant antitumor potential against human breast cancer by inhibiting the TLR4/MyD88/NF-κB (Lu et al., 2019) and MAPK pathway (Zhou et al., 2020) *in vitro* (Chen et al., 2017) and *in vivo*. However, the potential targets and pharmacological mechanism underlying the regulation of DMDD in lung cancer tumorigenesis still remain unclear. In this study, we predicted 60 DMDD potential target proteins via PharmMapper. These proteins were found to be highly involved in specific cellular processes such as protein binding, regulation of response to stimulus and various signaling pathways especially pathways in cancer, cell cycle and small cell lung cancer. These data suggested that cancer related genes and pathways were abundant in DMDD potential targets. In order to find the genes associated with lung cancer in DMDD potential targets, we analyzed the TCGA lung cancer dataset and found 8 DMDD potential therapeutic target candidates from 3,545 differently expressed genes in lung cancer. They were *FUT8, CCNE1, E2F1, ARHGAP11A, ARHGEF7, RUNX1T1, ACADVL* and *FES*. Among them, cell cycle related oncogenes *CCNE1* (Moroy and Geisen, 2004) and *E2F1* (Poppy Roworth et al., 2015) were high expression and predictive of poor patient survival in lung cancer. Moreover, we constructed a molecular docking model of DMDD and revealed that DMDD exhibited significant binding affinities within the active site of CCNE1 and E2F1. Furthermore, *in vitro* study showed that DMDD treatment significantly inhibits the proliferation, clone formation and migration of lung cancer cells H1975 and PC9. These results suggest that DMDD can inhibit the growth of lung cancer cells, and its mechanism may related to targeting CCNE1, E2F1 and further modulating cell cycle progression.

Cell cycle is a series of biological processes that takes place in a cell leading to its division and duplication (Martinez-Alonso and Malumbres, 2020). Abnormal cell cycle arrest may result in tumorigenesis (Petroni et al., 2020) and chemoresistance (Dokumcu and Farahani, 2019). DMDD potential targets CCNE1 (Ohtsubo et al., 1995) and E2F1 (Sahin and Sladek, 2010) are key regulator of the G1/S phase cell cycle transition. CCNE1 accumulates at the G1-S phase and degrades as cells progress through S phase. CCNE1 forms a complex with CDK2 and acts as a regulatory subunit of CDK2 (Honda et al., 2005). The CCNE1–CDK2 complex phosphorylates pRb, enhancing the E2F1 mediated expression of genes required for DNA synthesis and cell cycle progression towards the S phase (Zacksenhaus et al., 1993; Sahin and Sladek, 2010). Thus, cell cycle errors are associated with alterations of CCNE1 and E2F1. Overexpression of CCNE1 or E2F1 is associated with chromosome instability, mitosis, apoptosis and contributes to tumorigenesis (Bell and Ryan, 2004). E2F1 overexpression acts as a growth-promoting factor and is associated with poor prognosis in non-small cell lung cancer (Gorgoulis et al., 2002; Sun et al., 2018). CCNE1 expression is upregulated and correlates with E2F1 status in high-grade neuroendocrine lung tumors (Salon et al., 2007). Therefore, CCNE1 and E2F1 have been confirmed to hold the potential to be diagnostic and prognostic markers in a variety of tumors, especially in lung cancer (Singh et al., 2018; Garcia-Martinez et al., 2020). In this study, we found that high expression of CCNE1 (log2FC > 1, *q* < 0.01) was presented in 18 of 31 cancer types. E2F1 was significantly high expressed (log2FC > 1, *q* < 0.01) in 22 of 31 cancer types in TCGA data set. It is worth noting that CCNE1 and E2F1 were high expression in lung cancer and predictive of poor patient survival. In fact, previous clinical studies also showed that CCNE1 and E2F1 have a tumor promoting effect in many cancers including lung cancer (Salon et al., 2007; Garcia-Martinez et al., 2020). These results suggest that DMDD potential targets CCNE1, E2F1 are involved in the development of various cancers and are closely related to cell cycle and tumorigenesis in lung cancer. Mechanistic studies showed that DMDD treatment induced G1/S cell cycle arrest by suppressing CCNE1, E2F1 and CDK2 and upregulating tumor suppressor gene p21 in lung cancer cells. Taken together, these results suggest that DMDD regulated the cell cycle by causing G1/S arrest through decreasing the expression of DMDD potential targets E2F1 and CCNE2.

## Conclusion

We performed network pharmacology and experimental evaluation to reveal the pharmacological mechanism of DMDD against lung cancer, and extracted 60 potential therapeutic targets by using network pharmacology. DMDD potential targets were found to be highly involved in specific cellular processes such as protein binding, regulation of response to stimulus and various signaling pathways especially in cancer, cell cycle and small cell lung cancer by GO and KEGG enrichment analyses. Among the 60 potential therapeutic targets, cell cycle related genes *CCNE1* and *E2F1* were high expression and predictive of poor patient survival in lung cancer. DMDD has the potential to be an effective agent against lung cancer by inhibiting proliferation, clone formation, migration and causing cell cycle arrest at G1/S through decreasing the expression of DMDD potential targets E2F1 and CCNE2. This study demonstrated the utility of network pharmacology combined with TCGA database analysis to uncover the potential targets and mechanism of natural products in cancer treatment.

## Data Availability

The original contributions presented in the study are included in the article/Supplementary Material, further inquiries can be directed to the corresponding authors.

## References

[B1] Alves-Silva J. M. Romane A. Efferth T. Salgueiro L. (2017). North African medicinal plants traditionally used in cancer therapy. Front. Pharmacol. 8, 383 10.3389/fphar.2017.00383 28694778 PMC5483438

[B2] Bell L. A. Ryan K. M. (2004). Life and death decisions by E2F-1. Cell Death Diff. 11, 137–142. 10.1038/sj.cdd.4401324 14526389

[B3] Biankin A. V. Piantadosi S. Hollingsworth S. J. (2015). Patient-centric trials for therapeutic development in precision oncology. Nature. 526, 361–370. 10.1038/nature15819 26469047

[B4] Chen C. Nong Z. Xie Q. He J. Cai W. Tang X. (2017). 2-Dodecyl-6-methoxycyclohexa-2,5-diene-1,4-dione inhibits the growth and metastasis of breast carcinoma in mice. Sci. Rep. 7, 6704 10.1038/s41598-017-07162-3 28751740 PMC5532290

[B5] Committee Z. B. E. (1999). Zhonghua Bencao. Shanghai: Shanghai Science and Technology Press 715.

[B6] Desai A. G. Qazi G. N. Ganju R. K. El-Tamer M. Singh J. Saxena A. K. (2008). Medicinal plants and cancer chemoprevention. Curr. Drug Metabol. 9, 581–591. 10.2174/138920008785821657 PMC4160808 18781909

[B7] Dokumcu K. Farahani R. M. (2019). Evolution of resistance in cancer: a cell cycle perspective. Front. Oncol. 9, 376 10.3389/fonc.2019.00376 31143706 PMC6520611

[B8] Gao Y. Huang R. Gong Y. Park H. S. Wen Q. Almosnid N. M. (2015). The antidiabetic compound 2-dodecyl-6-methoxycyclohexa-2,5-diene-1,4-dione, isolated from Averrhoa carambola L., demonstrates significant antitumor potential against human breast cancer cells. Oncotarget 6, 24304–24319. 10.18632/oncotarget.4475 26203774 PMC4695187

[B9] Garcia-Martinez A. Lopez-Munoz B. Fajardo C. Camara R. Lamas C. Silva-Ortega S. (2020). Increased E2F1 mRNA and miR-17-5p expression is correlated to invasiveness and proliferation of pituitary neuroendocrine tumours. Diagnostics 10, 227 10.3390/diagnostics10040227 PMC7235816 32316225

[B10] Gillet J. P. Macadangdang B. Fathke R. L. Gottesman M. M. Kimchi-Sarfaty C. (2009). The development of gene therapy: from monogenic recessive disorders to complex diseases such as cancer. Methods Mol. Biol. 542, 5–54. 10.1007/978-1-59745-561-9_1 19565894

[B11] Gorgoulis V. G. Zacharatos P. Mariatos G. Kotsinas A. Bouda M. Kletsas D. (2002). Transcription factor E2F-1 acts as a growth-promoting factor and is associated with adverse prognosis in non-small cell lung carcinomas. J. Pathol. 198, 142–156. 10.1002/path.1121 12237873

[B12] Gyorffy B. Surowiak P. Budczies J. Lanczky A. (2013). Online survival analysis software to assess the prognostic value of biomarkers using transcriptomic data in non-small-cell lung cancer. PloS One 8, e82241 10.1371/journal.pone.0082241 24367507 PMC3867325

[B13] Hallstrom T. C. Nevins J. R. (2003). Specificity in the activation and control of transcription factor E2F-dependent apoptosis. Proc. Natl. Acad. Sci. USA. 100, 10848–10853. 10.1073/pnas.1831408100 12954980 PMC196891

[B14] Honda R. Lowe E. D. Dubinina E. Skamnaki V. Cook A. Brown N. R. (2005). The structure of cyclin E1/CDK2: implications for CDK2 activation and CDK2-independent roles. EMBO J. 24, 452–463. 10.1038/sj.emboj.7600554 15660127 PMC548659

[B15] Hopkins A. L. (2008). Network pharmacology: the next paradigm in drug discovery. Nat. Chem. Biol. 4, 682–690. 10.1038/nchembio.118 18936753

[B16] Keith C. T. Borisy A. A. Stockwell B. R. (2005). Multicomponent therapeutics for networked systems. Nat. Rev. Drug Discov. 4, 71–78. 10.1038/nrd1609 15688074

[B17] Kibble M. Saarinen N. Tang J. Wennerberg K. Makela S. Aittokallio T. (2015). Network pharmacology applications to map the unexplored target space and therapeutic potential of natural products. Nat. Prod. Rep. 32, 1249–1266. 10.1039/c5np00005j 26030402

[B18] Kintoko K. Xu X. Lin X. Jiao Y. Wen Q. Chen Z. (2018). Hypoglycaemic activity of 2-dodecyl-6-methoxycyclohexa-2,5-diene-1,4-dione in streptozotocin-induced diabetic mice through ameliorating metabolic function and regulating peroxisome proliferator-activated receptor gamma. Arch. Med. Sci. AMS. 14, 1163–1172. 10.5114/aoms.2016.63285 30154901 PMC6111351

[B19] Kowalczyk A. Jassem J. (2020). Multidisciplinary team care in advanced lung cancer. Transl. Lung Cancer Res. 9, 1690–1698. 10.21037/tlcr.2019.11.33 32953542 PMC7481611

[B20] Li Z. Q. Yan H. C. Gu J. J. Yang Y. L. Fang X. J. Zhang M. K. (2020). Comparative efficacy and safety of PD-1/PD-L1 Inhibitors versus platinum-based chemotherapy for the first-line treatment of advanced non-small cell lung cancer: a meta analysis of randomized controlled trials. Pharmacol. Res. 20, 105194 10.1016/j.phrs.2020.105194 32937178

[B21] Liu C. Li H. Wang K. Zhuang J. Chu F. Gao C. (2019). Identifying the antiproliferative effect of Astragalus polysaccharides on breast cancer: coupling network pharmacology with targetable screening from the cancer Genome Atlas. Front. Oncol. 9, 368 10.3389/fonc.2019.00368 31157164 PMC6533882

[B22] Liu X. F. Ouyang S. S. Yu B. A. Liu Y. B. Huang K. Gong J. Y. (2010). PharmMapper server: a web server for potential drug target identification using pharmacophore mapping approach. Nucl. Acids Res. 38, W609–W614. 20430828 10.1093/nar/gkq300 PMC2896160

[B23] Lu S. Zhang H. Wei X. Huang X. Chen L. Jiang L. (2019). 2-dodecyl-6-methoxycyclohexa-2,5-diene-1,4-dione isolated from Averrhoa carambola L. root ameliorates diabetic nephropathy by inhibiting the TLR4/MyD88/NF-kappaB pathway. Diabetes Metab. Syndr. Obes. Targets Therap. 12, 1355–1363. 10.2147/DMSO.S209436 PMC6689538 31496773

[B24] Martinez-Alonso D. Malumbres M. (2020). Mammalian cell cycle cyclins. Semin. Cell Dev. Biol. 107, 28–35. 10.1016/j.semcdb.2020.03.009 32334991

[B25] Moroy T. Geisen C. (2004). Cyclin E. Int. J. Biochem. Cell Biol. 36, 1424–1439. 10.1016/j.biocel.2003.12.005 15147722

[B26] Motoi N. Szoke J. Riely G. J. Seshan V. E. Kris M. G. Rusch V. W. (2008). Lung adenocarcinoma: modification of the 2004 WHO mixed subtype to include the major histologic subtype suggests correlations between papillary and micropapillary adenocarcinoma subtypes, EGFR mutations and gene expression analysis. Am. J. Surg. Pathol. 32, 810–827. 10.1097/PAS.0b013e31815cb162 18391747

[B27] Nagy A. Lanczky A. Menyhart O. Gyorffy B. (2018). Validation of miRNA prognostic power in hepatocellular carcinoma using expression data of independent datasets. Sci Rep-Uk. 8, 9227. 10.1038/s41598-018-27521-y PMC6003936 29907753

[B28] Newman D. J. Cragg G. M. (2016). Natural products as sources of new drugs from 1981 to 2014. J. Nat. Prod. 79, 629–661. 10.1021/acs.jnatprod.5b01055 26852623

[B29] Ohtsubo M. Theodoras A. M. Schumacher J. Roberts J. M. Pagano M. (1995). Human cyclin E, a nuclear protein essential for the G1-to-S phase transition. Mol. Cell Biol. 15, 2612–2624. 10.1128/mcb.15.5.2612 7739542 PMC230491

[B30] Ouyang L. Luo Y. Tian M. Zhang S. Y. Lu R. Wang J. H. (2014). Plant natural products: from traditional compounds to new emerging drugs in cancer therapy. Cell Prolif. 47, 506–515. 10.1111/cpr.12143 25377084 PMC6496831

[B31] Petroni G. Formenti S. C. Chen-Kiang S. Galluzzi L. (2020). Immunomodulation by anticancer cell cycle inhibitors. Nat. Rev. Immunol. 20 (11), 669–679. 10.1038/s41577-020-0300-y 32346095 PMC7584736

[B32] Poornima P. Kumar J. D. Zhao Q. Blunder M. Efferth T. (2016). Network pharmacology of cancer: from understanding of complex interactomes to the design of multi-target specific therapeutics from nature. Pharmacol. Res. 111, 290–302. 10.1016/j.phrs.2016.06.018 27329331

[B33] Poppy Roworth A. Ghari F. La Thangue N. B. (2015). To live or let die - complexity within the E2F1 pathway. Mol. Cell Oncol. 2, e970480 10.4161/23723548.2014.970480 27308406 PMC4905241

[B34] Sahin F. Sladek T. L. (2010). E2F-1 has dual roles depending on the cell cycle. Int. J. Biol. Sci. 6, 116–128. 10.7150/ijbs.6.116 20224733 PMC2836542

[B35] Salon C. Merdzhanova G. Brambilla C. Brambilla E. Gazzeri S. Eymin B. (2007). E2F-1, Skp2 and cyclin E oncoproteins are upregulated and directly correlated in high-grade neuroendocrine lung tumors. Oncogene. 26, 6927–6936. 10.1038/sj.onc.1210499 17471231

[B36] Scagliotti G. V. Parikh P. von Pawel J. Biesma B. Vansteenkiste J. Manegold C. (2008). Phase III study comparing cisplatin plus gemcitabine with cisplatin plus pemetrexed in chemotherapy-naive patients with advanced-stage non-small-cell lung cancer. J. Clin. Oncol. Off. J. Am. Soc. Clin. Oncol. 26, 3543–3551. 10.1200/JCO.2007.15.0375 18506025

[B37] Seca A. M. L. Pinto D. (2018). Plant secondary metabolites as anticancer agents: successes in clinical trials and therapeutic application. Int. J. Mol. Sci. 19, 263 10.3390/ijms19010263 PMC5796209 29337925

[B38] Shannon P. Markiel A. Ozier O. Baliga N. S. Wang J. T. Ramage D. (2003). Cytoscape: a software environment for integrated models of biomolecular interaction networks. Genome Res. 13, 2498–2504. 14597658 10.1101/gr.1239303 PMC403769

[B39] Siegel R. L. Miller K. D. Jemal A. (2020). Cancer statistics, 2020. CA Cancer J. Clin. 70, 7–30. 31912902 10.3322/caac.21590

[B40] Singh S. Gupta M. Sharma A. Seam R. K. Changotra H. (2018). The nonsynonymous polymorphisms Val276Met and Gly393Ser of E2F1 gene are strongly associated with lung, and head and neck cancers. Genet. Test. Mol. Biomark. 22, 498–502. 10.1089/gtmb.2018.0066 30036075

[B41] Sterling T. Irwin J. J. (2015). ZINC 15-ligand discovery for everyone. J. Chem. Inf. Model 55, 2324–2337. 26479676 10.1021/acs.jcim.5b00559 PMC4658288

[B42] Sun C. C. Zhou Q. Hu W. Li S. J. Zhang F. Chen Z. L. (2018). Transcriptional E2F1/2/5/8 as potential targets and transcriptional E2F3/6/7 as new biomarkers for the prognosis of human lung carcinoma. Aging. 10, 973–987. 10.18632/aging.101441 29754146 PMC5990399

[B43] Tariq A. Sadia S. Pan K. Ullah I. Mussarat S. Sun F. (2017). A systematic review on ethnomedicines of anti-cancer plants. Phytother. Res. PTR. 31, 202–264. 10.1002/ptr.5751 28093828

[B44] Wang X. Pan C. X. Gong J. Y. Liu X. F. Li H. L. (2016). Enhancing the enrichment of pharmacophore-based target prediction for the polypharmacological profiles of drugs. J. Chem. Inf. Model 56, 1175–1183. 27187084 10.1021/acs.jcim.5b00690

[B45] Wang X. Shen Y. H. Wang S. W. Li S. L. Zhang W. L. Liu X. F. (2017). PharmMapper 2017 update: a web server for potential drug target identification with a comprehensive target pharmacophore database. Nucl. Acids Res. 45, W356–W360. 28472422 10.1093/nar/gkx374 PMC5793840

[B46] Wen Q. Lin X. Liu Y. Xu X. Liang T. Zheng N. (2012). Phenolic and lignan glycosides from the butanol extract of Averrhoa carambola L. root. Molecules 17, 12330–12340. 10.3390/molecules171012330 23085667 PMC6268650

[B47] Yao H. P. Hudson R. Wang M. H. (2020). Progress and challenge in development of biotherapeutics targeting MET receptor for treatment of advanced cancer. Biochim. Biophys. Acta Rev. Cancer 18, 8425 10.1016/j.bbcan.2020.188425 32961258

[B48] Youlden D. R. Cramb S. M. Baade P. D. (2008). The International Epidemiology of Lung Cancer: geographical distribution and secular trends. J. Thorac. Oncol. Off. Publ. Int. Assoc. Study Lung Cancer 3, 819–831. 10.1097/JTO.0b013e31818020eb 18670299

[B49] Zacksenhaus E. Bremner R. Phillips R. A. Gallie B. L. (1993). A bipartite nuclear localization signal in the retinoblastoma gene product and its importance for biological activity. Mol. Cell. Biol. 13, 4588–4599. 10.1128/mcb.13.8.4588 8336704 PMC360081

[B50] Zhang R. Zhu X. Bai H. Ning K. (2019). Network pharmacology databases for traditional Chinese medicine: review and assessment. Front. Pharmacol. 10, 123 10.3389/fphar.2019.00123 30846939 PMC6393382

[B51] Zhou X. Wu X. Qin L. Lu S. Zhang H. Wei J. (2020). Anti-breast cancer effect of 2-dodecyl-6-methoxycyclohexa-2,5-diene-1,4-dione *in vivo* and *in vitro* through MAPK signaling pathway. Drug Des. Dev. Therap. 14, 2667–2684. 10.2147/DDDT.S237699 PMC7369253 32764871

[B52] Zhou Y. Y. Zhou B. Pache L. Chang M. Khodabakhshi A. H. Tanaseichuk O. (2019). Metascape provides a biologist-oriented resource for the analysis of systems-level datasets. Nat. Commun. 10, 1523 10.1038/s41467-019-09234-6 30944313 PMC6447622

