# Supplementary material for: Uncovering the Pharmacological Mechanism of 2-Dodecyl-6-Methoxycyclohexa-2,5 -Diene-1,4-Dione Against Lung Cancer Based on Network Pharmacology and Experimental Evaluation
Source: Front Pharmacol. 2021 Feb 2;12:617555. doi: 10.3389/fphar.2021.617555 (PMC7887632; doi:10.3389/fphar.2021.617555)
Supplement: Supplementary file 2 [file presentation1.pptx]

## Slide 1
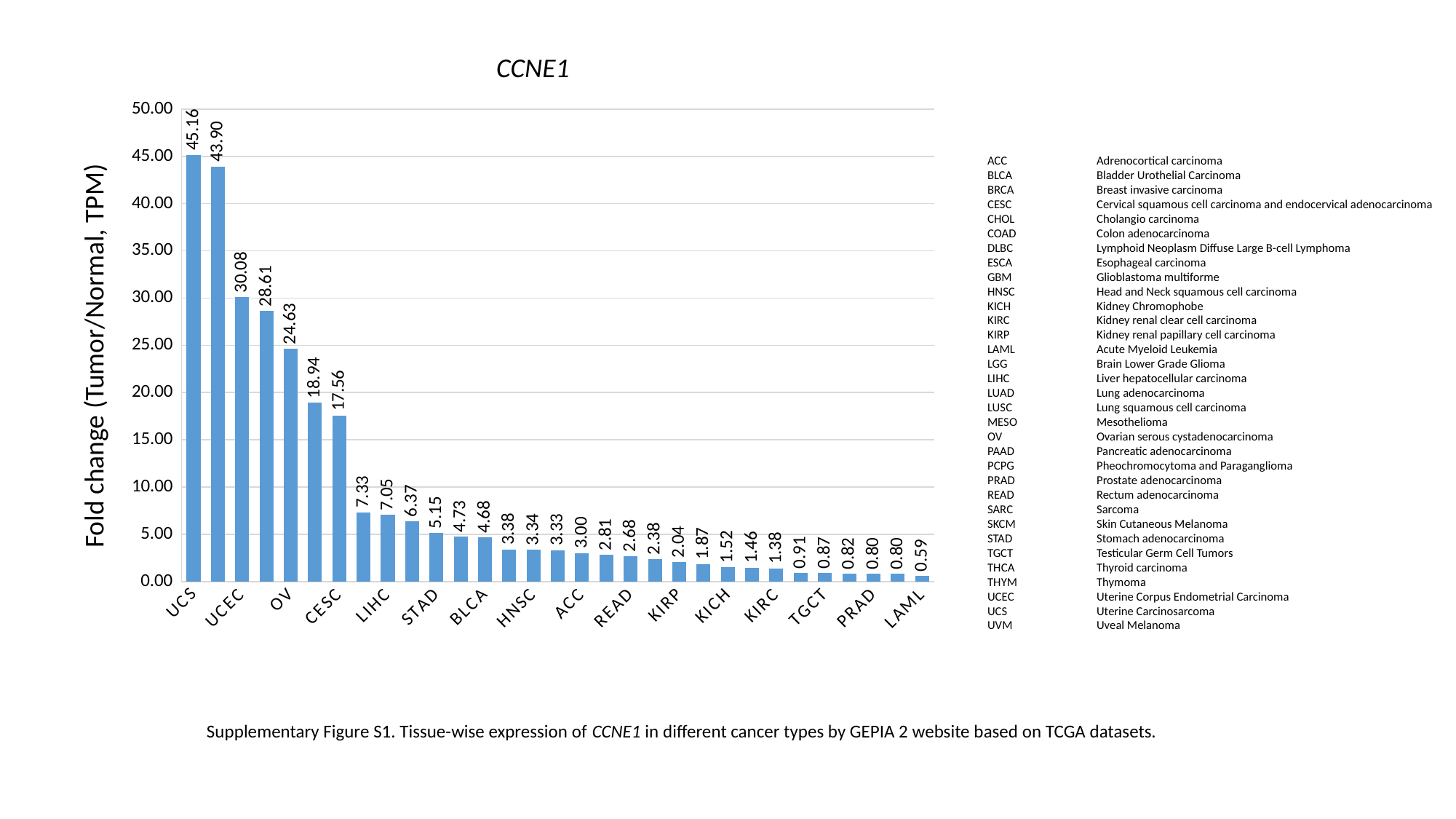

CCNE1
### Chart
| Category | |
|---|---|
| UCS | 45.16216216216217 |
| DLBC | 43.90322580645161 |
| UCEC | 30.076923076923077 |
| CHOL | 28.611111111111114 |
| OV | 24.630952380952383 |
| THYM | 18.939393939393938 |
| CESC | 17.564102564102562 |
| LUSC | 7.330578512396694 |
| LIHC | 7.045454545454546 |
| PAAD | 6.36842105263158 |
| STAD | 5.152671755725191 |
| SARC | 4.733333333333333 |
| BLCA | 4.679245283018868 |
| LUAD | 3.3781512605042017 |
| HNSC | 3.339285714285714 |
| BRCA | 3.333333333333333 |
| ACC | 2.9965635738831615 |
| COAD | 2.808080808080808 |
| READ | 2.684385382059801 |
| ESCA | 2.3762626262626263 |
| KIRP | 2.0422535211267605 |
| SKCM | 1.8668478260869565 |
| KICH | 1.5157894736842106 |
| GBM | 1.4565916398713827 |
| KIRC | 1.3766233766233766 |
| THCA | 0.9069767441860466 |
| TGCT | 0.8741653826399589 |
| LGG | 0.8167202572347267 |
| PRAD | 0.803030303030303 |
| PCPG | 0.7982456140350878 |
| LAML | 0.5870930896630496 |ACC	Adrenocortical carcinoma
BLCA	Bladder Urothelial Carcinoma
BRCA	Breast invasive carcinoma
CESC	Cervical squamous cell carcinoma and endocervical adenocarcinoma
CHOL	Cholangio carcinoma
COAD	Colon adenocarcinoma
DLBC	Lymphoid Neoplasm Diffuse Large B-cell Lymphoma
ESCA	Esophageal carcinoma
GBM	Glioblastoma multiforme
HNSC	Head and Neck squamous cell carcinoma
KICH	Kidney Chromophobe
KIRC	Kidney renal clear cell carcinoma
KIRP	Kidney renal papillary cell carcinoma
LAML	Acute Myeloid Leukemia
LGG	Brain Lower Grade Glioma
LIHC	Liver hepatocellular carcinoma
LUAD	Lung adenocarcinoma
LUSC	Lung squamous cell carcinoma
MESO	Mesothelioma
OV	Ovarian serous cystadenocarcinoma
PAAD	Pancreatic adenocarcinoma
PCPG	Pheochromocytoma and Paraganglioma
PRAD	Prostate adenocarcinoma
READ	Rectum adenocarcinoma
SARC	Sarcoma
SKCM	Skin Cutaneous Melanoma
STAD	Stomach adenocarcinoma
TGCT	Testicular Germ Cell Tumors
THCA	Thyroid carcinoma
THYM	Thymoma
UCEC	Uterine Corpus Endometrial Carcinoma
UCS	Uterine Carcinosarcoma
UVM	Uveal Melanoma
Fold change (Tumor/Normal, TPM)
Supplementary Figure S1. Tissue-wise expression of CCNE1 in different cancer types by GEPIA 2 website based on TCGA datasets.

## Slide 2
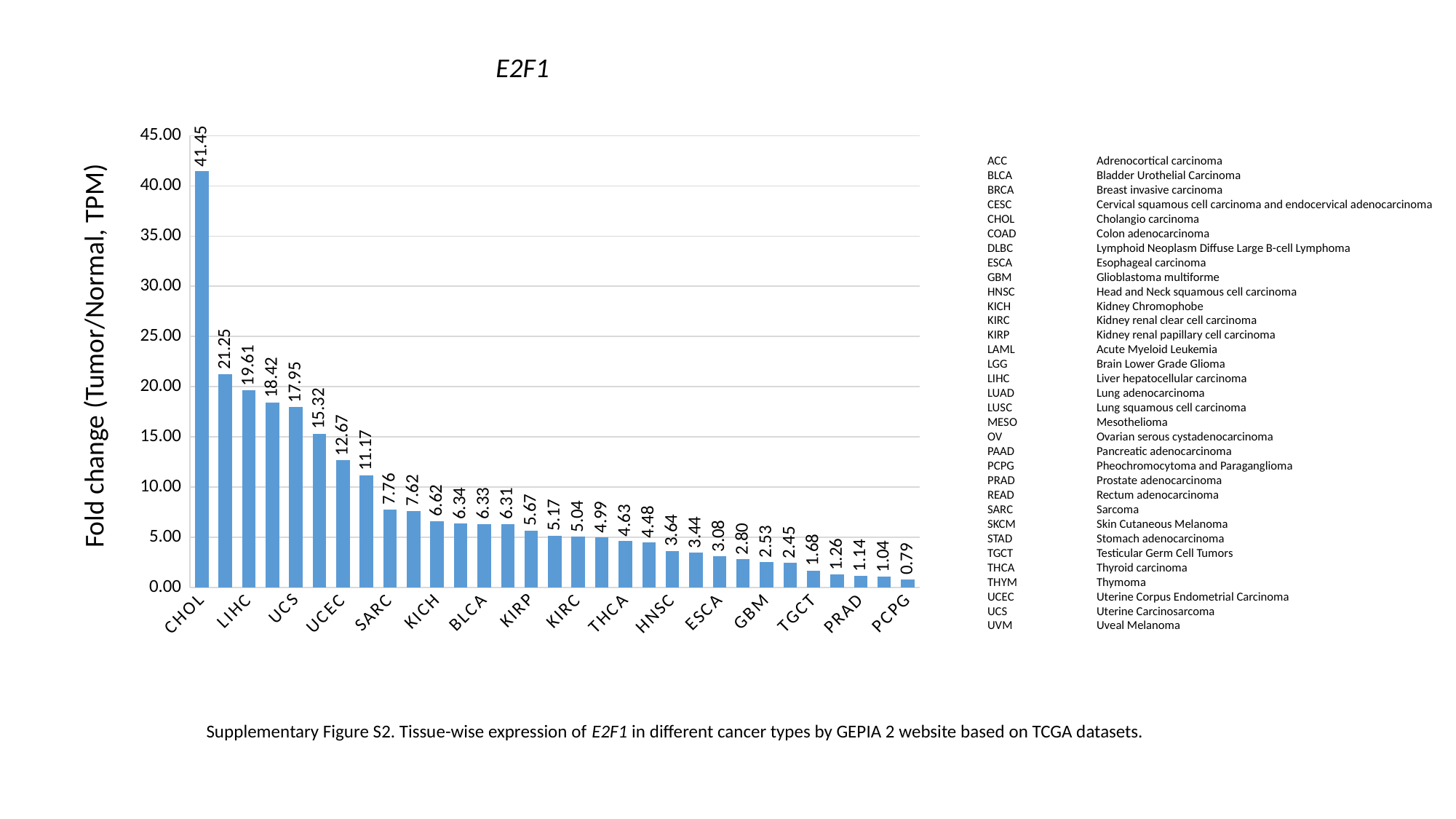

E2F1
### Chart
| Category | |
|---|---|
| CHOL | 41.45454545454545 |
| DLBC | 21.245454545454546 |
| LIHC | 19.607142857142858 |
| CESC | 18.42063492063492 |
| UCS | 17.94545454545454 |
| PAAD | 15.32142857142857 |
| UCEC | 12.669642857142856 |
| THYM | 11.17272727272727 |
| SARC | 7.75503355704698 |
| READ | 7.616915422885573 |
| KICH | 6.619047619047619 |
| COAD | 6.342723004694836 |
| BLCA | 6.331606217616581 |
| BRCA | 6.3125 |
| KIRP | 5.666666666666666 |
| STAD | 5.172774869109948 |
| KIRC | 5.037735849056603 |
| OV | 4.993690851735016 |
| THCA | 4.629213483146067 |
| SKCM | 4.483425414364641 |
| HNSC | 3.6440677966101696 |
| LUSC | 3.440729483282675 |
| ESCA | 3.081841432225064 |
| ACC | 2.8014888337468977 |
| GBM | 2.53125 |
| LUAD | 2.4524495677233427 |
| TGCT | 1.684055841293167 |
| LAML | 1.2623985572587917 |
| PRAD | 1.1436464088397789 |
| LGG | 1.041015625 |
| PCPG | 0.7876288659793814 |ACC	Adrenocortical carcinoma
BLCA	Bladder Urothelial Carcinoma
BRCA	Breast invasive carcinoma
CESC	Cervical squamous cell carcinoma and endocervical adenocarcinoma
CHOL	Cholangio carcinoma
COAD	Colon adenocarcinoma
DLBC	Lymphoid Neoplasm Diffuse Large B-cell Lymphoma
ESCA	Esophageal carcinoma
GBM	Glioblastoma multiforme
HNSC	Head and Neck squamous cell carcinoma
KICH	Kidney Chromophobe
KIRC	Kidney renal clear cell carcinoma
KIRP	Kidney renal papillary cell carcinoma
LAML	Acute Myeloid Leukemia
LGG	Brain Lower Grade Glioma
LIHC	Liver hepatocellular carcinoma
LUAD	Lung adenocarcinoma
LUSC	Lung squamous cell carcinoma
MESO	Mesothelioma
OV	Ovarian serous cystadenocarcinoma
PAAD	Pancreatic adenocarcinoma
PCPG	Pheochromocytoma and Paraganglioma
PRAD	Prostate adenocarcinoma
READ	Rectum adenocarcinoma
SARC	Sarcoma
SKCM	Skin Cutaneous Melanoma
STAD	Stomach adenocarcinoma
TGCT	Testicular Germ Cell Tumors
THCA	Thyroid carcinoma
THYM	Thymoma
UCEC	Uterine Corpus Endometrial Carcinoma
UCS	Uterine Carcinosarcoma
UVM	Uveal Melanoma
Fold change (Tumor/Normal, TPM)
Supplementary Figure S2. Tissue-wise expression of E2F1 in different cancer types by GEPIA 2 website based on TCGA datasets.

## Slide 3
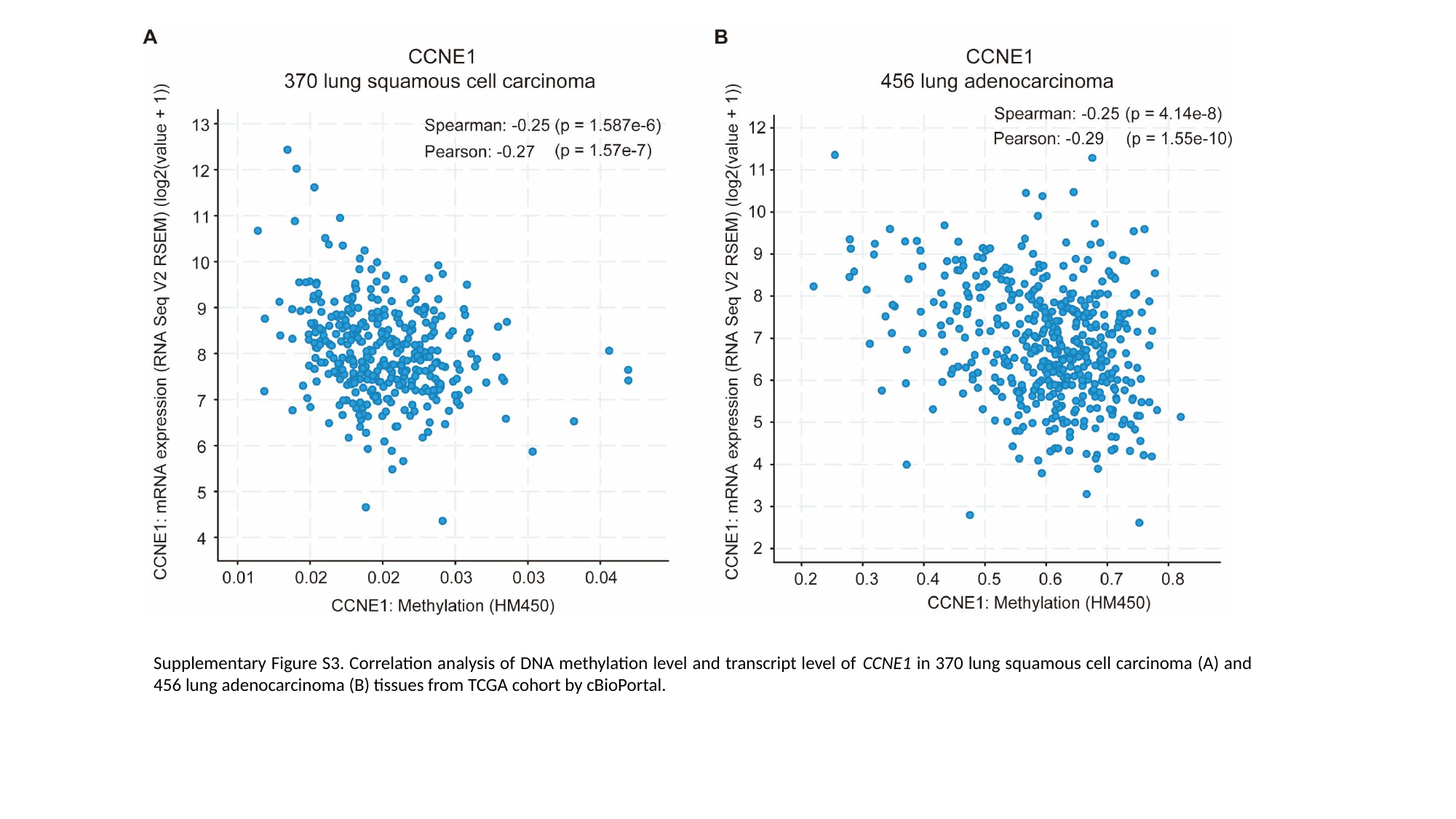

Supplementary Figure S3. Correlation analysis of DNA methylation level and transcript level of CCNE1 in 370 lung squamous cell carcinoma (A) and 456 lung adenocarcinoma (B) tissues from TCGA cohort by cBioPortal.

## Slide 4
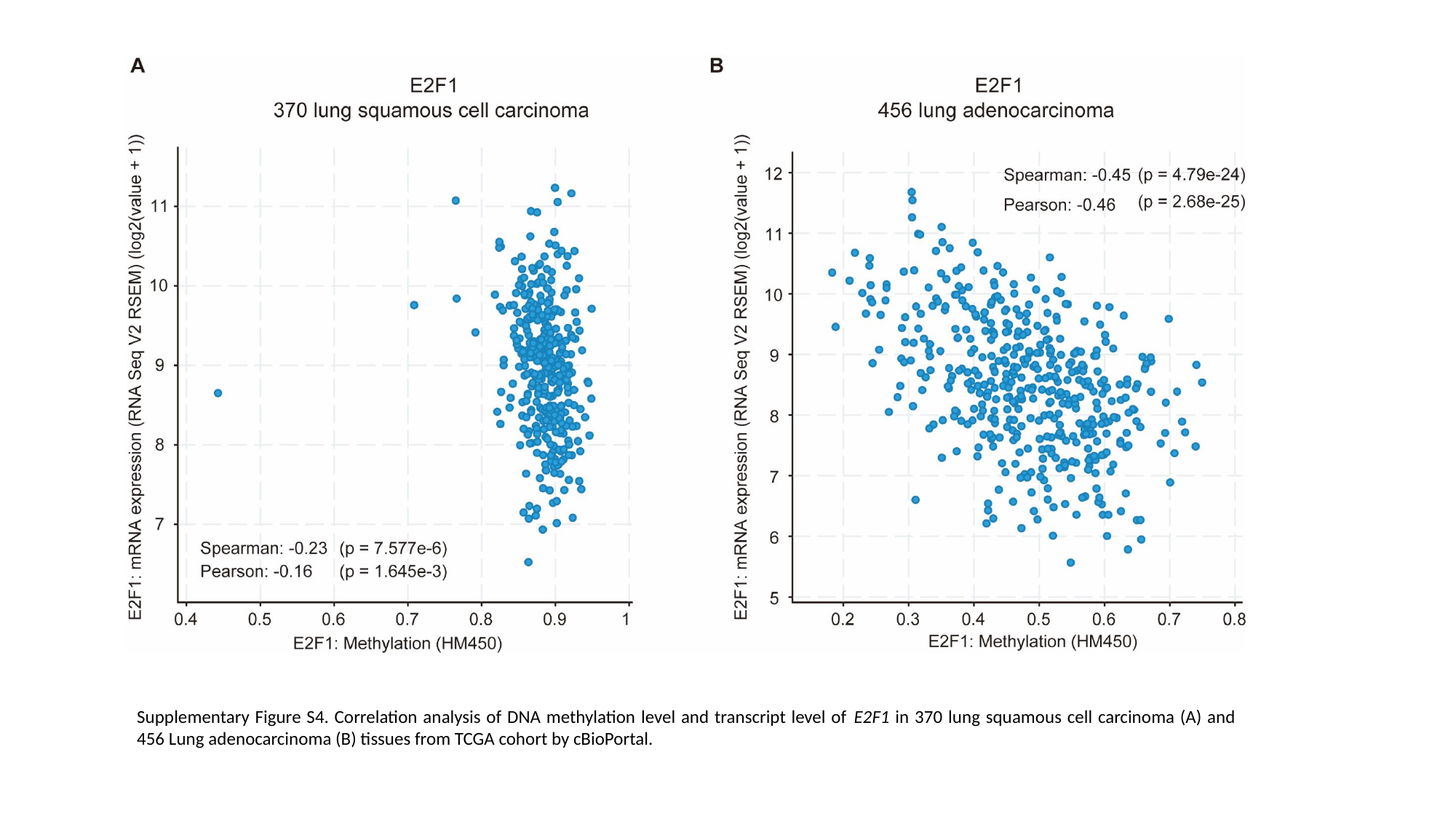

Supplementary Figure S4. Correlation analysis of DNA methylation level and transcript level of E2F1 in 370 lung squamous cell carcinoma (A) and 456 Lung adenocarcinoma (B) tissues from TCGA cohort by cBioPortal.

## Slide 5
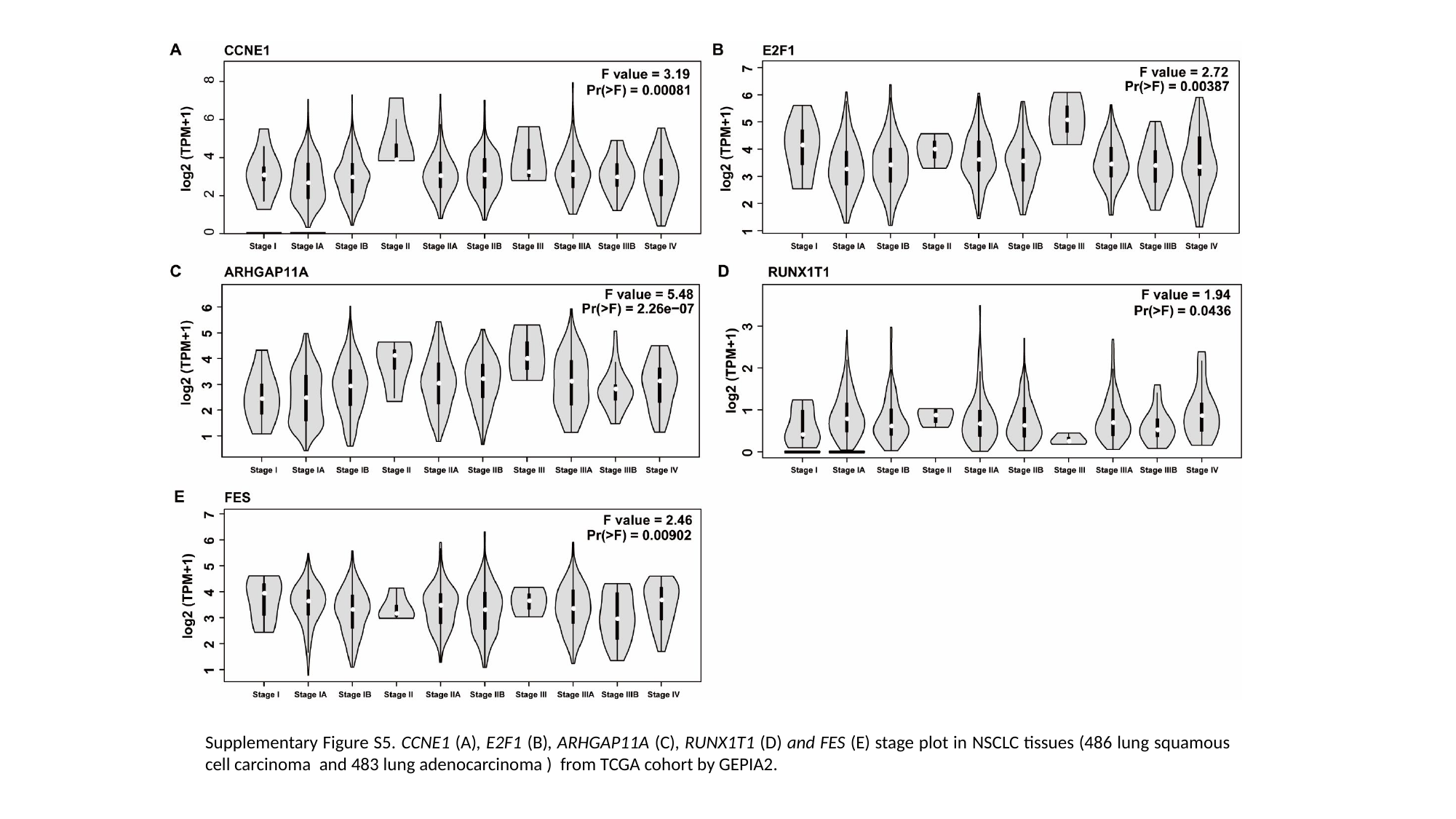

Supplementary Figure S5. CCNE1 (A), E2F1 (B), ARHGAP11A (C), RUNX1T1 (D) and FES (E) stage plot in NSCLC tissues (486 lung squamous cell carcinoma and 483 lung adenocarcinoma ) from TCGA cohort by GEPIA2.
